# The Diagnostic Value of bpMRI in Prostate Cancer: Benefits and Limitations Compared to mpMRI

**DOI:** 10.3390/bioengineering11101006

**Published:** 2024-10-09

**Authors:** Roxana Iacob, Diana Manolescu, Emil Robert Stoicescu, Simona Cerbu, Răzvan Bardan, Laura Andreea Ghenciu, Alin Cumpănaș

**Affiliations:** 1 Doctoral School, ‘Victor Babes’ University of Medicine and Pharmacy Timisoara, Eftimie Murgu Square No. 2, 300041 Timisoara, Romania; roxana.iacob@umft.ro; 2 Department of Anatomy and Embriology, ‘Victor Babes’ University of Medicine and Pharmacy Timisoara, Eftimie Murgu Square No. 2, 300041 Timisoara, Romania; 3 Research Center for Medical Communication, ‘Victor Babes’ University of Medicine and Pharmacy Timisoara, Eftimie Murgu Square No. 2, 300041 Timisoara, Romania; 4 Field of Applied Engineering Sciences, Specialization Statistical Methods and Techniques in Health and Clinical Research, Faculty of Mechanics, ‘Politehnica’ University Timisoara, Mihai Viteazul Boulevard No. 1, 300222 Timisoara, Romania; 5 Department of Radiology and Medical Imaging, ‘Victor Babes’ University of Medicine and Pharmacy Timisoara, Eftimie Murgu Square No. 2, 300041 Timisoara, Romania; dmanolescu@umft.ro (D.M.); cerbu.simona@umft.ro (S.C.); 6 Research Center for Pharmaco-Toxicological Evaluations, ‘Victor Babes’ University of Medicine and Pharmacy Timisoara, Eftimie Murgu Square No. 2, 300041 Timisoara, Romania; 7 Department of Urology, ‘Victor Babes’ University of Medicine and Pharmacy Timisoara, Eftimie Murgu Square No. 2, 300041 Timisoara, Romania; bardan.razvan@umft.ro (R.B.); cumpanas.alin@umft.ro (A.C.); 8 Department of Functional Sciences, ‘Victor Babes’ University of Medicine and Pharmacy Timisoara, Eftimie Murgu Square No. 2, 300041 Timisoara, Romania; bolintineanu.laura@umft.ro

**Keywords:** mpMRI, bpMRI, prostate cancer imaging, prostate cancer diagnosis, prostate volume assessment

## Abstract

Prostate cancer is the second most common cancer in men and a leading cause of death worldwide. Early detection is vital, as it often presents with vague symptoms such as nocturia and poor urinary stream. Diagnostic tools like PSA tests, ultrasound, PET-CT, and mpMRI are essential for prostate cancer management. The PI-RADS system helps assess malignancy risk based on imaging. While mpMRI, which includes T1, T2, DWI, and dynamic contrast-enhanced imaging (DCE), is the standard, bpMRI offers a contrast-free alternative using only T2 and DWI. This reduces costs, acquisition time, and the risk of contrast-related side effects but has limitations in detecting higher-risk PI-RADS 3 and 4 lesions. This study compared bpMRI’s diagnostic accuracy to mpMRI, focusing on prostate volume and PI-RADS scoring. Both methods showed strong inter-rater agreement for prostate volume (ICC 0.9963), confirming bpMRI’s reliability in this aspect. However, mpMRI detected more complex conditions, such as periprostatic fat infiltration and iliac lymphadenopathy, which bpMRI missed. While bpMRI offers advantages like reduced cost and no contrast use, it is less effective for higher-risk lesions, making mpMRI more comprehensive.

## 1. Introduction

Prostate cancer represents the second most common cancer diagnosis in men and the fifth greatest cause of death globally [1]. Unfortunately, in the early stages, prostate cancer is often asymptomatic or gives unspecific symptoms, such as nocturia, poor urinary stream, erectile dysfunctions, and hematuria [2]. Despite having an indolent course that may simply require active surveillance (AS), some prostate cancers are aggressive and require curative treatment or palliative measures (if found in late stages, which are associated with metastases across the organism) [1,3].

Prostate-specific antigen (PSA) is a protein produced by prostate cells, and its levels are measured through a blood test. Elevated PSA levels can indicate various prostate issues, including benign conditions like prostate enlargement, benign prostate hyperplasia (BPH), or prostatitis, as well as more serious conditions like prostate cancer [4,5]. While high PSA levels are a concern, they do not always mean cancer is present, as factors like age or recent prostate exams can also cause an increase [5]. 

Early diagnosis of prostate cancer is essential for achieving optimal patient outcomes. Common imaging modalities used for screening, diagnosis, and monitoring include active surveillance (AS), prostate ultrasound (US), PET-CT, and multiparametric MRI (mpMRI) [6,7].

**Figure 1:**
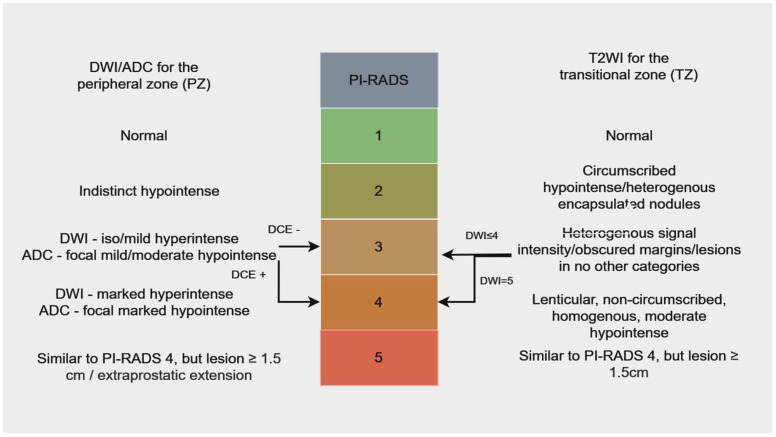
PI−RADS v2 for mpMRI, adapted from Lo et al. [9]—Colors were used to differentiate various prostate conditions, with green representing the normal prostate, which has a very low cancer risk. Benign lesions, with low to intermediate cancer risk, were indicated by a darker shade of green and a bright shade of orange. High-risk lesions were marked in dark orange, while red signified lesions with a very high likelihood of being malignant.

For an objective report of the prostatic lesion, radiologists use the Prostate Imaging Reporting and Data System (PI-RADS). This score helps radiologists and clinicians understand how likely it is that a suspicious zone is benign or malignant. The most recent version of PI-RADS—namely v2.1—was launched in 2019, and contains specific performance adjustments, easing some recommendations that were shown to be ineffective [8]. This scoring system helps the clinician in making the right decision regarding the management of prostate lesions. The following figure is a summarized explanation of this score (Figure 1).

In recent years, mpMRI has become the most routinely utilized imaging modality for examining the prostate [10]. Sequences like T1-weighted, T2-weighted, diffusion-weighted (DWI), and dynamic contrast-enhanced imaging (DCE) each provide unique information about tissues or organs. Depending on the suspected pathology, these sequences are used together, complementing each other to aid in accurate diagnosis [11,12]. T1-weighted (T1W) sequences are common in MRI protocols and provide images that closely resemble the tissue’s macroscopic appearance. T2-weighted imaging (T2WI) is a basic MRI sequence that highlights differences in tissue T2 relaxation times [13]. Diffusion-weighted imaging (DWI) measures water movement in tissues, revealing details about cellularity, tumors, and edema. DCE provides structural and functional insights by analyzing tissue enhancement after gadolinium contrast injection [14]. The use of contrast agents and the DCE sequence has drawbacks, including contrast allergies, nephropathy in patients with low glomerular filtration rates, higher costs, and longer acquisition times [15].

Due to the drawbacks of mpMRI, bpMRI (without contrast and the DCE sequence) has gained attention. It avoids contrast-induced adverse reactions, lowers costs, and shortens acquisition time by using only two sequences—T2-weighted imaging (T2WI) and diffusion-weighted imaging (DWI). This makes it more cost-effective and safer [16].

However, bpMRI has limitations compared to mpMRI. The contrast-enhanced sequences in mpMRI offer additional information on tissue vascularization, improving specificity in detecting suspicious lesions and evaluating prostate cancer recurrence [14].

The above-presented sequences and their explanations can be found in the table below (Table 1).

MRI machines are categorized by magnetic field strength, measured in Tesla (T), named after inventor Nikola Tesla. Tesla quantifies the strength of the MRI scanner’s magnetic field, with higher values indicating stronger fields and better image quality. For example, a 1.5 T MRI scanner has a field strength of 1.5 Tesla, while a 3.0 T scanner has a field strength of 3.0 Tesla, improving image detail by aligning more protons in the body’s tissues [17].

The prostate gland is divided into four zones: the largest, the peripheral zone (PZ); the central zone (CZ), which accounts for 25% of the glandular tissue; the transition zone (TZ); and the anterior fibromuscular stroma [18]. The majority of PCa (up to 70%) is found in the PZ, while 20% can be found in the TZ. In the CZ, just 1% of this neoplasia develops [19].

**Figure 2:**
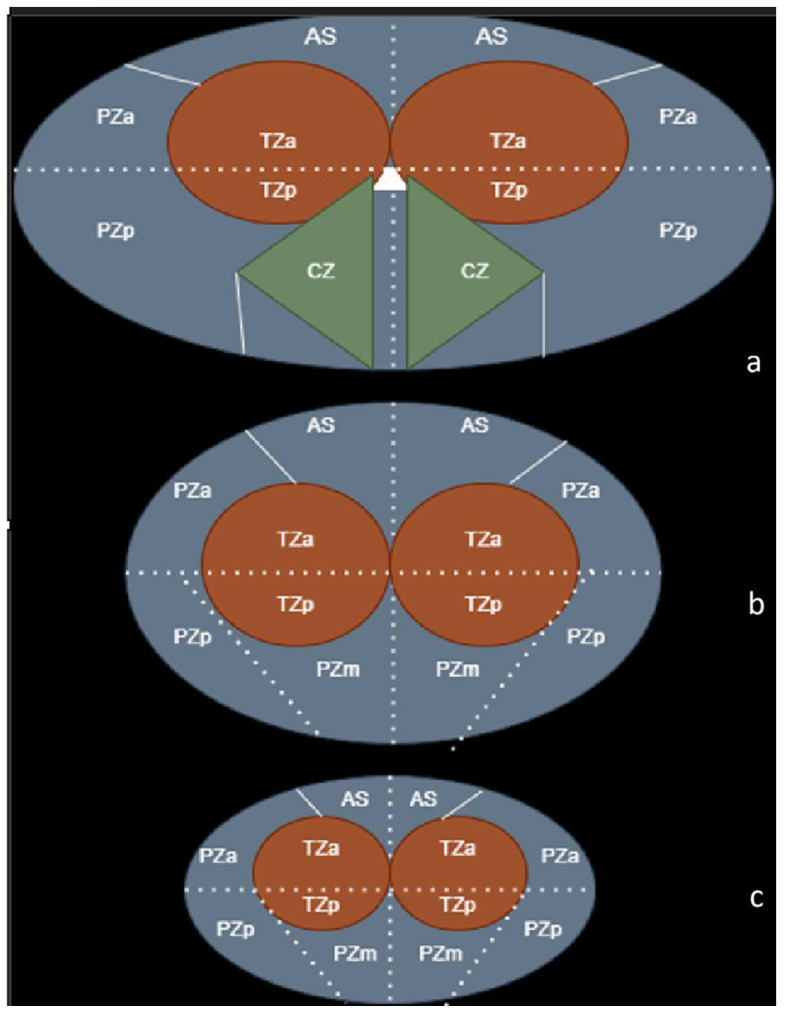
Anatomy of the prostate, adapted by Radiology Assistant [20]—zones (**a**)—base, (**b**)—middle segment, (**c**)—apex. AS—anterior fibromuscular stroma; TZ(a/p)—anterior and posterior transition zone; PZ(a/m)—anterior and posterior peripheric zone; PZm—the right and left posteromedial peripheral zone.

The following picture is a schematic representation of the prostate gland, for a better understanding of the sectional anatomy (Figure 2).

This research highlights the novelty of bpMRI as a cost-effective, non-invasive alternative to mpMRI in prostate cancer diagnosis. Our study aims to demonstrate that bpMRI provides comparable accuracy in prostate volume measurements and PI-RADS scoring, offering reliable results without the need for contrast agents, thus reducing patient risk and lowering costs.

## 2. Materials and Methods

### 2.1. Patients’ Selection

Prostate mpMRIs of patients scanned at ‘Dr. Victor Babes’ Hospital of Infectious Diseases and Pneumophtisiology Timisoara, Romania, over a period of 3 years (January 2020–November 2023) were selected. From these, the studies that belonged to men under 40 years old, and incomplete MRIs were excluded from the study. MpMRI studies of men with previous prostate neoplasia or interventions on the prostate or periprostatic tissue were also excluded. After implementing the exclusion criteria, 92 prostate mpMRIs were eligible for the study.

The MRI machine used for the acquisition of the images was a Signa Explorer 1.5 Tesla, designed and manufactured by GE Healthcare. The machine is equipped with high-performance gradient coils, allowing for rapid switching of magnetic field gradients. Its software allows for customization of imaging protocols and offers advanced post-processing tools for image manipulation and visualization.

T2 and DWI sequences were examined on a lot of 92 prostate MRIs; DWI was used as the primary sequence in the peripheral zone, while T2 was used as the primary sequence in the transverse zone. These imaging studies were scored by two independent experienced radiologists (one with 7 years’ experience on prostate MRI, and the other with 12 years of experience), who had no contact with the original mpMRI report, PI-RADS score, and PSA of the patients. The bpMRI of the two readers’ (Reader 1 and Reader 2) scores were compared to the PI-RADS score of the original interpretation. Additionally, the ellipsoid volumes of the prostate were evaluated by both bpMRI readers, and the results were compared with the initial report. The ellipsoid volume formula is the following [21]:

Ellipsoid volume = L × W × H × π/6

**Figure 3:**
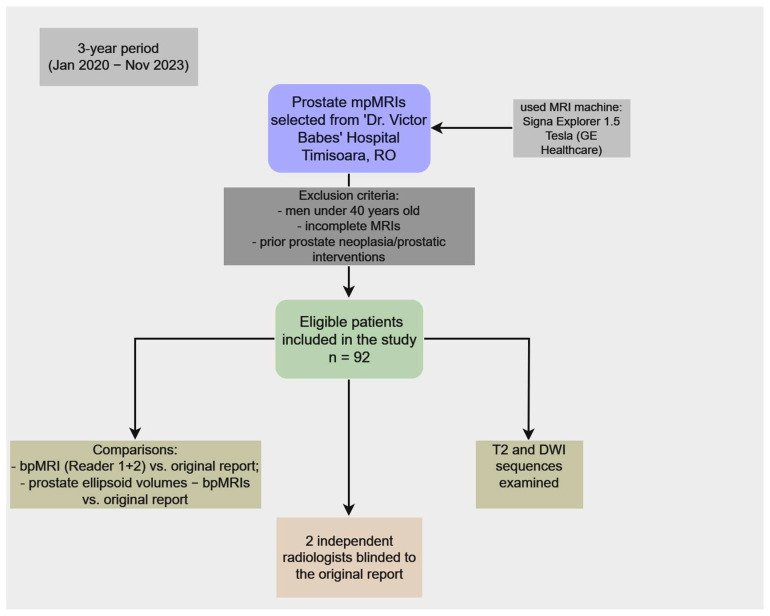
Flowchart illustrating the design of our study.

The following flowchart represents the explanation of the study design (Figure 3).

The study was conducted and approved by the Ethics Committee of ‘Dr. Victor Babes’ Hospital of Infectious Diseases and Pneumophtisiology Timisoara, Romania (approval number 7914/06.09.2024).

### 2.2. Statistical Analysis

The statistical analysis was performed using MedCalc^®^ Statistical Software version 22.016 64-bit (MedCalc Software Ltd., Ostend, Belgium). Descriptive statistics were calculated for all continuous variables and are presented as medians with interquartile ranges (IQRs) or arithmetic means with standard deviations (SDs), depending on the distribution of the data. Categorical variables were expressed as frequencies and percentages.

The sample size for this study was determined based on the availability of patients who underwent both mpMRI and bpMRI during the study period. A total of 92 patients were included, ensuring a representative sample of individuals requiring prostate imaging for assessment of prostate volume and cancer risk using the PI-RADS scoring system. Given the high reliability of prostate volume measurements and PI-RADS scoring, the sample size was deemed sufficient to provide robust estimates of inter-rater agreement and consistency, as reflected by the intraclass correlation coefficient (ICC) and kappa statistics. Additionally, the chosen sample size aligns with previous studies evaluating inter-rater reliability in prostate imaging, allowing for accurate detection of meaningful differences across raters.

To assess inter-rater reliability and agreement, both the ICC and weighted kappa statistics were employed. ICCs were used to measure the consistency and absolute agreement among raters for continuous variables such as prostate ellipsoid volume. Both single and average measures ICCs were calculated, with corresponding 95% confidence intervals. A value of ICC greater than 0.75 was considered indicative of excellent agreement.

For categorical assessments, such as the PI-RADS scoring system, weighted kappa coefficients were calculated to assess inter-rater agreement. Quadratic weights were used for prostate ellipsoid volume measurements, and linear weights were applied for the PI-RADS classification. Kappa values were interpreted as follows: values between 0.81 and 1.00 indicated almost perfect agreement, 0.61 to 0.80 substantial agreement, 0.41 to 0.60 moderate agreement, 0.21 to 0.40 fair agreement, and values less than 0.20 indicated slight agreement.

The distribution of measurements between raters was visually inspected using violin plots, box plots, and individual data points to explore differences between mpMRI and bpMRI. Additionally, for categorical variables such as PI-RADS, bar plots were employed to demonstrate the frequency distribution across different categories and raters. The Friedman test was used to compare PI-RADS scores from mpMRI and bpMRI (Readers 1 and 2) for cases with mpMRI scores of 3 and 4. Statistical significance was set at a *p*-value < 0.05.

## 3. Results

### 3.1. Descriptive Statistics

Out of a total of 92 patients included, 55.43% resided in metropolitan locations. The table below provides an overview of key demographic, anatomical, and diagnostic parameters relevant to prostate assessment. The analyzed parameters are presented in Table 2 (Summary of Patient Characteristics and Prostate Imaging Parameters) as median/arithmetic mean and IQR or standard deviation.

The median age of the patients was 69 years, with an interquartile range of 62–74 years. Prostate ellipsoid volumes measured by mpMRI and bpMRI across two readers show consistent median values of approximately 41 mL, with slight variations in IQR. The PI-RADS scores, used for assessing prostate cancer risk, show a median score of 2 across both mpMRI and bpMRI readings. Lastly, prostate-specific antigen levels have a median value of 10.25 ng/mL.

### 3.2. Interclass Correlation Coefficient and Inter-Rater Agreement (Kappa)

**Figure 4:**
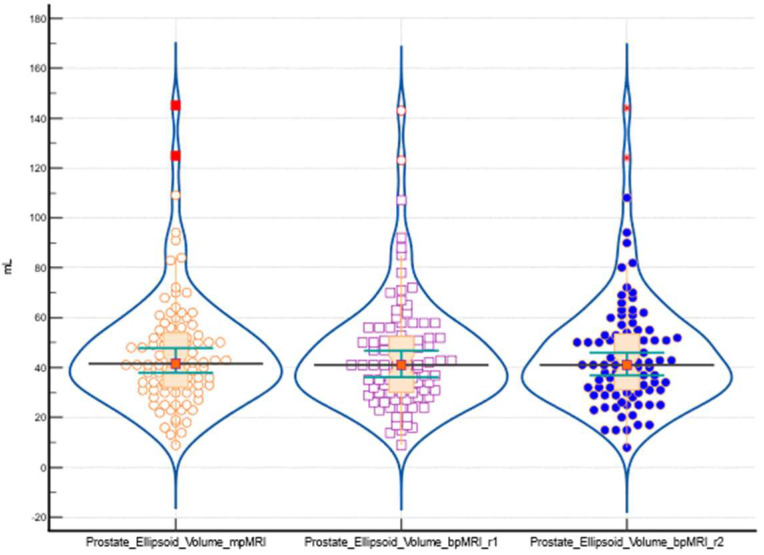
The distribution of prostate ellipsoid volume measured using both mpMRI and bpMRI using a combination of violin plots, box plots, and individual data points. Each violin plot represents the density of the data within the respective group, with wider sections indicating a higher concentration of values. Overlaid within each violin is a box plot, highlighting the IQR, with a central line denoting the median value and whiskers extending to capture the spread of the data. Outliers are visible as individual points outside the whiskers. In addition to these visual summaries, individual data points are superimposed on the plot, represented by orange circles for the first group, purple squares for the second group, and blue dots for the third group.

The intraclass correlation coefficient (ICC) for absolute agreement among the three raters measuring prostate ellipsoid volume showed a single measures ICC of 0.9963 with a 95% confidence interval of 0.9945 to 0.9976. The ICC for average measures was 0.9988, with a 95% confidence interval of 0.9982 to 0.9992. These values indicate the degree of agreement among the raters for individual and averaged measurements using both mpMRI and bpMRI methods. Figure 4 presents the distribution graph with differences between prostate ellipsoid volume measured using both mpMRI and bpMRI.

**Figure 5:**
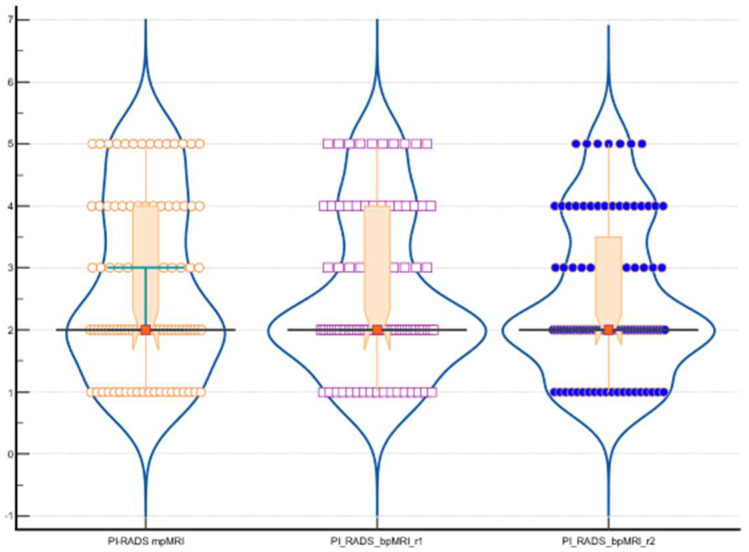
The distribution of PI-RADS using both mpMRI and bpMRI using a combination of violin plots, box plots, and individual data points. Each violin plot represents the density of the data within the respective group, with wider sections indicating a higher concentration of values. Overlaid within each violin is a box plot, highlighting the IQR, with a central line denoting the median value and whiskers extending to capture the spread of the data. Outliers are visible as individual points outside the whiskers. In addition to these visual summaries, individual data points are superimposed on the plot, represented by orange circles for the first group, purple squares for the second group, and blue dots for the third group.

The ICC for absolute agreement among the three raters measuring PI-RADS scores showed a single measures ICC of 0.9070 with a 95% confidence interval of 0.8692 to 0.9354. The ICC for average measures was 0.9670, with a 95% confidence interval of 0.9522 to 0.9775. These values indicate the level of agreement among the raters for individual and averaged PI-RADS score measurements using both mpMRI and bpMRI methods. Figure 5 presents the distribution of the PI-RADS, highlighting differences between measurements obtained using mpMRI and bpMRI across different raters.

The inter-rater agreement for prostate ellipsoid volume measured by bpMRI shows a weighted kappa of 0.9927 with a standard error of 0.00182 and a 95% confidence interval (CI) of 0.98912 to 0.99627, using quadratic weights. This indicates almost perfect agreement between raters in measuring prostate volume, reflecting excellent reliability.

For the PI-RADS assessment measured by bpMRI, the weighted kappa is 0.79754, with a standard error of 0.03263 and a 95% CI of 0.73358 to 0.86149, calculated using linear weights. This suggests substantial agreement between raters, though slightly lower than for prostate volume measurements. These are presented in Table 3.

**Figure 6:**
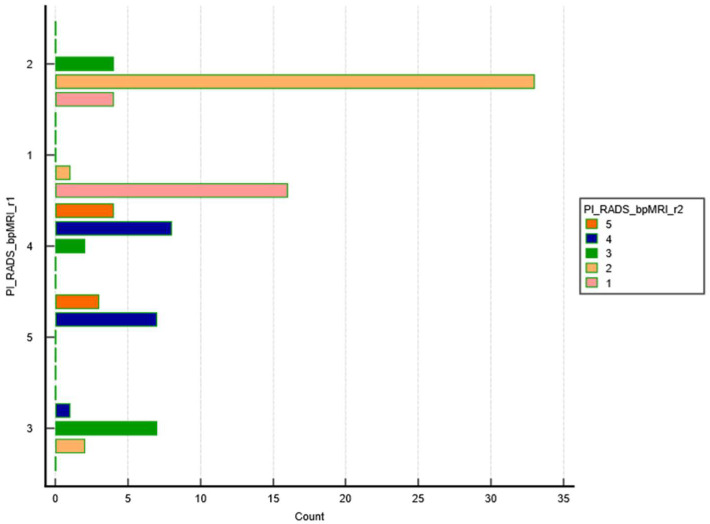
The graph illustrates the distribution of PI-RADS scores as assessed by Readers 1 and 2 using bpMRI (biparametric MRI). The *y*-axis lists the different PI-RADS categories (from 1 to 5), while the *x*-axis represents the frequency of cases in each category. Each color in the bar represents a specific PI-RADS score: pink for PI-RADS 1, yellow for PI-RADS 2, green for PI-RADS 3, blue for PI-RADS 4, orange for PI-RADS 5.

Figure 6 shows the distribution of PI-RADS bpMRI ratings by Reader 2. Most cases are rated as PI-RADS 2, followed by smaller groups in PI-RADS 1 and PI-RADS 3, with fewer cases in PI-RADS 4 and 5. The majority of cases are rated as PI-RADS 2, as represented by the longest yellow bar, indicating that most subjects were classified as having a lower risk of clinically significant prostate cancer. A smaller but notable proportion of cases were rated as PI-RADS 1 (low risk) and PI-RADS 3, while fewer cases fell into the PI-RADS 4 and PI-RADS 5 categories, which represent higher risk.

This chart visually represents the distribution of PI-RADS scores for the cohort, showing that most patients were assessed as low risk, with relatively few falling into the higher-risk categories.

### 3.3. Comparison of PI-RADS Classifications between mpMRI and bpMRI for Scores of 3 and 4

In this analysis of 13 cases where the PI-RADS mpMRI score was consistently 3, a significant difference was observed between the PI-RADS classifications provided by mpMRI and bpMRI (Friedman test, *p* = 0.009). The descriptive statistics show that both bpMRI readers assigned lower scores, with a median PI-RADS of 2 and an IQR of 2 to 3. In contrast, mpMRI classified all cases with a PI-RADS score of 3. The multiple comparisons indicate that mpMRI consistently assigned significantly higher scores compared to both bpMRI, Reader 1, and Reader 2, with mean ranks of 2.57 for mpMRI, 1.65 for bpMRI Reader 1, and 1.76 for bpMRI Reader 2. There was no statistically significant difference between the two bpMRI readers. This suggests that mpMRI systematically classified patients at a higher risk level (PI-RADS 3) compared to bpMRI, highlighting potential discrepancies in prostate cancer risk assessment between the two imaging modalities.

In this analysis of 15 cases where the PI-RADS mpMRI score was consistently 4, no statistically significant difference was observed between the PI-RADS classifications provided by mpMRI and bpMRI, as indicated by the Friedman test (*p* = 0.093). The descriptive statistics show that both bpMRI readers generally assigned similar scores, with a median PI-RADS of 4 for both, though bpMRI Reader 1 ranged from 3 to 4 and bpMRI Reader 2 ranged from 3 to 5. In contrast, mpMRI consistently classified all cases with a PI-RADS score of 4. The Friedman test resulted in a non-significant difference (F = 2.57), suggesting that the PI-RADS scores between the methods (mpMRI, bpMRI Reader 1, and bpMRI Reader 2) were relatively similar in this group of cases. This indicates less discrepancy in prostate cancer risk assessment between mpMRI and bpMRI when the mpMRI score is 4, as compared to cases where mpMRI scores are lower, such as 3. There was no significant difference between the bpMRI readers’ classifications.

**Figure 7:**
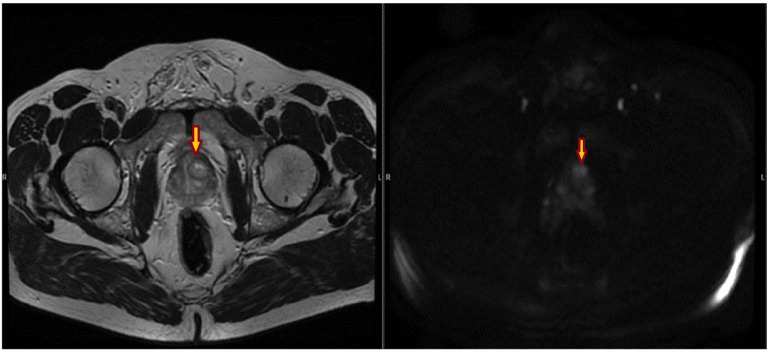
T2 and DWI sequences, showing an uncircumscribed lesion, with restricted diffusion, localized in the anterior transitional zone (left)—yellow arrow. The lesion was scored PI-RADS 5 by all of the radiologists.

**Figure 8:**
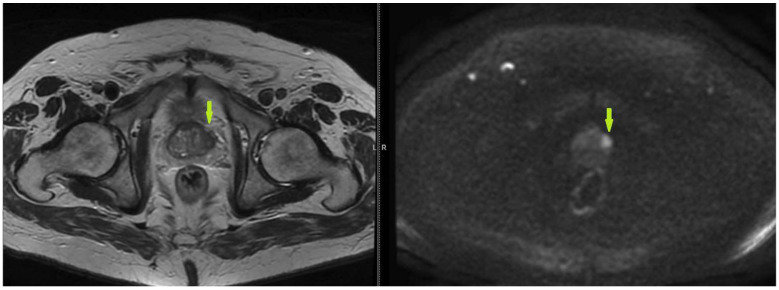
T2 and DWI sequences of a prostate MRI, showing a lenticular lesion, uncircumscribed, with T2 hyposignal and restricted diffusion, localized in the transitional left zone, with peripheric extension—scored PI-RADS 5 (green arrow).

The following images show different suspicious lesions, seen both in bpMRI and mpMRI (Figure 7 and Figure 8).

## 4. Discussion

Our study’s findings on prostate ellipsoid volume measurement align with previous research that underscores the reliability of mpMRI and bpMRI for prostate volume assessment. The high inter-rater agreement in our study, demonstrated by an ICC of 0.9963 for prostate volume, is consistent with results from other studies that have reported similarly high inter-rater agreement for volume measurements using these imaging modalities. Other studies also found excellent inter-rater reliability in prostate volume assessment using mpMRI, with ICC values exceeding 0.95, supporting the robustness of these methods for clinical evaluation [22,23].

Additionally, our results indicate a high level of agreement for PI-RADS scoring between raters, with an ICC of 0.9070 for single measures. This is in line with other studies that have examined PI-RADS inter-rater variability. Rosenkrantz et al. demonstrated substantial agreement between radiologists for PI-RADS scores using mpMRI, though they noted slightly higher variability when bpMRI was used [24]. Our findings, especially regarding lower kappa values for PI-RADS assessment using bpMRI (0.79754), similarly reflect this challenge, suggesting that bpMRI may introduce slightly more variability in PI-RADS evaluation than mpMRI.

Moreover, the median PSA level in our cohort (10.25 ng/mL) is comparable to levels seen in other studies focused on prostate cancer screening using MRI. Other studies highlight similar PSA values in cohorts undergoing MRI for prostate cancer diagnosis, supporting the representativeness of our patient population [25].

BpMRI tends to yield lower PI-RADS 3 and 4 scores compared to mpMRI, indicating that while bpMRI is a reliable method, mpMRI may provide more detailed assessment, particularly for higher-risk lesions. This could suggest that mpMRI is superior in identifying both intermediate and high-risk lesions, making it more effective for comprehensive prostate cancer evaluation. These results align with other studies, which suggest that bpMRI’s limitations in distinguishing between PI-RADS 3 and 4 lesions highlight the importance of using mpMRI for a more comprehensive evaluation and accurate characterization of suspicious prostate lesions, especially when a finer distinction between these categories is required for clinical decision-making [26,27].

When comparing our study to other research in the field, similar trends are evident. In a study conducted by Westphalen et al., it was highlighted that mpMRI tends to classify more lesions as clinically significant (PI-RADS 3), which aligns with our findings of mpMRI assigning higher scores in intermediate-risk cases. In another study, however, Asif et al. found bpMRI to sometimes outperform mpMRI in detecting clinically significant prostate cancer, especially for PI-RADS 3 lesions, supporting bpMRI’s more conservative risk stratification, as seen in our study. Both studies also found comparable performance between bpMRI and mpMRI for high-risk cases like PI-RADS 4, reflecting our observations of similar accuracy across modalities for detecting aggressive cancers [28,29].

Regarding associated changes and pathologies in the pelvic area, the comparison between bpMRI and mpMRI shows that while both methods effectively identify common pathologies such as thickened bladder walls, diverticulosis, hydroceles, and encondromas, mpMRI excels in detecting more complex conditions. MpMRI was able to identify additional findings like periprostatic fat infiltration, iliac lymphadenopathy, rectal duplication cysts, and perianal fistulas, which were not detected by bpMRI. Furthermore, mpMRI provided more detailed information on vascular and seminal vesicle abnormalities, likely due to its use of contrast-enhanced sequences. These findings suggest that while bpMRI is effective for detecting common pelvic conditions, mpMRI offers more comprehensive evaluations, especially for complex and multi-organ pathologies, likely due to its contrast-enhanced imaging capabilities.

As evidenced by the current study’s findings and other specialized research in the field, bpMRI has several advantages compared to MRI with contrast substance—mpMRI [16,30].

Since it only uses two sequences, as its name implies, bpMRI has an easier assessment, making it faster to comprehend and potentially permitting quicker and more accurate radiological examinations. Thus, the radiologist requires only two sequences that he has to study to make a presumptive diagnosis, and studies show that many doctors prefer this type of investigation compared to mpMRI [16,30]. Even though it lacks some of the information offered by mpMRI, bpMRI can nevertheless provide a targeted assessment of anatomical structures, assisting in the diagnosis and localization of significant prostate lesions. Additionally, given the result of our study, as well as other studies on this topic, bpMRI has the possibility to diagnose and evaluate other changes associated or unrelated to prostate pathology, detectable in the pelvic cavity [31]. Thus, bpMRI can evaluate changes in soft tissues in the pelvic cavity, and in the gastrointestinal tract.

Regarding MRI contrast agents, studies have shown that adverse effects from gadolinium contrast agents are infrequent, with a reported prevalence of 1.5–2.4% [32,33]. Most adverse effects are regarded as mild, or moderate, and they most typically include urticaria, vasodilatation, mucosal reactions, vomiting, change in taste, local warmth, local pain, headache, paresthesia, and dizziness [32,33,34].

In assessing the prostate with bpMRI instead of mpMRI, the choice between bullet prostate volume and ellipsoid volume depends on factors like ease of measurement, accuracy, and the specific diagnostic requirements. Bullet prostate volume, simpler to measure with transverse and longitudinal dimensions, aligns well with the streamlined approach of bpMRI. On the other hand, ellipsoid volume offers geometric accuracy but involves more complex calculations [35]. The decision should consider the balance between simplicity and accuracy in line with bpMRI’s efficiency goals. Factors such as available resources, team expertise, and imaging study goals also influence the choice. Ultimately, selecting the volume measurement method in bpMRI involves careful consideration of these factors to ensure reliable and clinically relevant assessments. As mentioned before, in our study, we chose to calculate the ellipsoid volume, as this method was the preferred approach used by the radiologist who initially interpreted the mpMRI.

A PI-RADS score above 2 indicates a higher risk of clinically significant prostate cancer, making it a valuable threshold for selecting individuals who may need further diagnostic evaluations, such as targeted biopsies or follow-up imaging. While scores of 1 or 2 are typically associated with low-risk or benign findings, higher scores suggest suspicious lesions that require closer examination [9]. This approach helps prioritize patients for additional testing, improving early detection and potentially leading to better outcomes in prostate cancer treatment and management.

Although bpMRI has limitations compared to mpMRI, its advantages could make it an important tool for potential prostate cancer screening. Its reduced cost, shorter acquisition time, and avoidance of contrast agents make it more accessible and safer for broader use. Implementing bpMRI in population-wide screening could help detect prostate cancer earlier, especially in resource-limited settings, while minimizing risks and increasing patient compliance. These benefits could contribute to more efficient and widespread prostate cancer screening, ultimately improving outcomes and reducing healthcare burdens.

As limitations of the study, we believe it is important to mention the fact that we did not have the chance yet to capture the long-term performance of bpMRI, due to the lack of follow-up studies. Another important limitation is the clinical heterogeneity of the studied lot, because their diverse comorbidities can make it hard to determine the impact of bpMRI regarding only prostate pathologies.

Some potential ideas regarding future research on this subject may include Artificial Intelligence software that can enhance the diagnostic capabilities of bpMRI in the management of prostate pathology. Additionally, prostate biopsy strategies or techniques could be developed to improve accuracy in the diagnosis of prostate cancer. While most of the studies are based on the diagnostic accuracy of prostate cancer by using bpMRI, long-term follow-up studies should be conducted to determine the applications and limitations of bpMRI over time. Finally, more studies regarding the patients’ perspective in terms of quality of life, anxiety, and satisfaction with bpMRI imaging in the management of prostatic pathologies should be conducted.

Further studies should explore the potential applications of bpMRI in AS for prostate cancer. Additionally, bpMRI could be valuable in assessing the effects of treatment in patients with known prostate cancer, providing crucial insights into disease progression or remission without the need for contrast agents, because many of them have contraindications of contrast administrations, due to the treatment’s effects (the failure of the renal function).

## 5. Conclusions

The study shows strong consistency between mpMRI and bpMRI in measuring prostate volume, with excellent inter-rater agreement (ICC 0.9963 for single measures and 0.9988 for averages). This supports bpMRI as a reliable alternative for prostate size assessment. While PI-RADS scores also showed good agreement, they exhibited slightly more variability, indicating room for improved standardization in cancer risk assessment. Overall, bpMRI offers a reliable, non-invasive option, reducing the need for contrast agents and associated costs. While bpMRI reduces patient discomfort and streamlines imaging, its limitations in differentiating PI-RADS 3 from 4 highlight the need for mpMRI for more accurate lesion characterization.

## Figures and Tables

**Table 1:** MRI sequences explanations.

MRI Sequence	Image Aquisition Principle	Information Provided
T1-weighted (T1)	Proton Density-based Image Acquisition–Measures tissue proton density.	It provides ‘anatomical’ images resembling macroscopic tissue appearances. Examples: Fat appears bright (high signal), while water and soft tissues appear darker (low signal).
T2-weighted (T2)	Emphasizes tissue T2 relaxation. The sequence highlights disparities in tissue T2 relaxation time, aiding in tissue characterization.	Highlights disparities in tissue T2 relaxation time, aiding in tissue characterization. Examples: Fluid-filled structures appear bright (high signal), while dense structures appear darker (low signal).
Diffusion-weighted (DWI)	Measures water molecule movement within tissues.	It reflects tissue cellularity, cell swelling, and edema. Examples: Tumors with high cellularity restrict water movement, appearing bright (high signal), while areas of edema or fluid accumulation may appear darker (low signal).
Dynamic contrast-enhanced (DCE)	Monitors tissue enhancement post-injection of contrast agent.	It offers morphological and functional information based on contrast uptake. Examples: Rapid contrast enhancement may indicate areas of increased vascularity or neovascularization, suggesting malignancy. Slower enhancement may be seen in benign conditions.

**Table 2:** Summary of patient characteristics and prostate imaging parameters.

Parameter	Median/Arithmetic Mean	IQR/SD
Age (years)	69	62–74
Prostate Ellipsoid Volume (mL) measured by mpMRI	41.50	32–54
Prostate Ellipsoid Volume (mL) measured by bpMRI—reader 1	41	30–52.50
Prostate Ellipsoid Volume (mL) measured by bpMRI—reader 2	41	31–53.50
PI-RADS mpMRI	2	2–4
PI-RADS bpMRI—reader 1	2	2–4
PI-RADS bpMRI—reader 2	2	2–3.5
Prostate-Specific Antigen (ng/mL)	10.25	4.85–21.05

**Table 3:** Inter-rater agreement for prostate ellipsoid volume and PI-RADS measured by bpMRI.

Parameter	Inter-Rater Agreement— Weighted Kappa	Standard Error	95% CI
Prostate Ellipsoid Volume (mL) measured by bpMRI	0.99270 ^a^	0.00182	0.98912 to 0.99627
PI-RADS bpMRI	0.79754 ^b^	0.03263	0.73358 to 0.86149

^a^—quadratic weights ^b^—linear weights.

## Data Availability

The information is contained within this article in its entirety. For additional information, please feel free to inquire with either the original author or the corresponding author. Public access to the data is restricted as a result of the patient privacy standards that regulate the handling of clinical data.
